# Supplementary material for: A preliminary survey of zoantharian endosymbionts shows high genetic variation over small geographic scales on Okinawa-jima Island, Japan
Source: PeerJ. 2017 Oct 3;5:e3740. doi: 10.7717/peerj.3740 (PMC5629959; doi:10.7717/peerj.3740)
Supplement: Supplemental Information 1 — Note not all specimens had their ITS2 sequences acquired as no clear distinctions asides from the two major groupings shown in Fig. 1A were found. [file peerj-05-3740-s001.docx]

Table S1. *Palythoa tuberculosa* specimens examined in this study, their collection information, and *Symbiodinium* GenBank Accession Numbers. Note not all specimens had their ITS2 sequences acquired as no clear distinctions asides from the two major groupings shown in Figure 1a were found.

| **Specimen** | **Depth (m)** | **Collection date** | **ITS2 GenBank Accession No.** | **ITS2 subclade**** | **psbA^ncr^ forward GenBank Accession No.** | **psbA^ncr^ forward lineage***** |
| --- | --- | --- | --- | --- | --- | --- |
| Wase-Amami 3 (A1)* | 1 to 2 | Jan. 27, 2012 |  |  | XXXX | 4 |
| Wase-Amami 6 (A6) | 1 to 2 | Jan. 27, 2012 | XXXX | C1 | XXXX | 1 |
| Wase-Amami 7 (A7) | 1 to 2 | Jan. 27, 2012 |  |  | XXXX | 1 |
| Wase-Amami 9 (A9) | 1 to 2 | Jan. 27, 2012 |  |  | XXXX | 1 |
| Wase-Amami 10 (A10) | 1 to 2 | Jan. 27, 2012 |  |  | XXXX | 1 |
| Oku 1 | 1 to 2 | Aug. 24, 2012 |  |  | XXXX | 2 |
| Oku 2 | 1 to 2 | Aug. 24, 2012 |  |  | XXXX | 2 |
| Oku 3 | 1 to 2 | Aug. 24, 2012 | XXXX | C1 | XXXX | 2 |
| Oku 4 | 1 to 2 | Aug. 24, 2012 |  |  | XXXX | 2 |
| Oku 5 | 1 to 2 | Aug. 24, 2012 |  |  | XXXX | 2 |
| Oku 6 | 1 to 2 | Aug. 24, 2012 |  |  | XXXX | 2 |
| Oku 7 | 1 to 2 | Aug. 24, 2012 | XXXX | C1 | XXXX | 2 |
| Oku 8 | 1 to 2 | Aug. 24, 2012 |  |  | XXXX | 1 |
| Oku 12 | 1 to 2 | Aug. 24, 2012 |  |  | XXXX | 1 |
| Oku 13 | 1 to 2 | Aug. 24, 2012 | XXXX | C1 | XXXX | 2 |
| Nerome 1 (N1) | 0 to 1 | Aug. 29, 2015 | XXXX | C1 | XXXX | 1 |
| Nerome 2 (N2) | 0 to 1 | Aug. 29, 2015 | XXXX | C1 | XXXX | 3 |
| Nerome 4 (N4) | 0 to 1 | Aug. 29, 2015 | XXXX | C1 | XXXX | 3 |
| Nerome 5 (N5) | 0 to 1 | Aug. 29, 2015 | XXXX | C1 | XXXX | 3 |
| Nerome 6 (N6) | 0 to 1 | Aug. 29, 2015 | XXXX | C1 | XXXX | 3 |
| Nerome 7 (N7) | 0 to 1 | Aug. 29, 2015 | XXXX | C1 | XXXX | 3 |
| Nerome 9 (N9) | 0 to 1 | Aug. 29, 2015 |  |  | XXXX | 1 |
| Nerome 10 (N10) | 0 to 1 | Aug. 29, 2015 | XXXX | C1-related | XXXX | 1 |
| Bise 1 (B1) | 0 to 1 | Nov. 30, 2015 |  |  | XXXX | 1 |
| Bise 2 (B2) | 0 to 1 | Nov. 30, 2015 | XXXX | C1 | XXXX | 3 |
| Bise 3 (B3) | 0 to 1 | Nov. 30, 2015 |  |  | XXXX | 1 |
| Bise 4 (B4)* | 0 to 1 | Nov. 30, 2015 |  |  | XXXX | 1 |
| Bise 5 (B5) | 0 to 1 | Nov. 30, 2015 |  |  | XXXX | 1 |
| Bise 8 (B8) | 0 to 1 | Nov. 30, 2015 |  |  | XXXX | 1 |
| Bise 13 (B13) | 0 to 1 | Nov. 30, 2015 |  |  | XXXX | 1 |
| Bise 14 (B14) | 0 to 1 | Nov. 30, 2015 | XXXX | C1 | XXXX | 3 |
| Bise 15 (B15) | 0 to 1 | Nov. 30, 2015 | XXXX | C1 | XXXX | 1 |
| Bise 17 (B17) | 0 to 1 | Nov. 30, 2015 |  |  | XXXX | 1 |
| Bise 19 (B19) | 0 to 1 | Nov. 30, 2015 |  |  | XXXX | 1 |
| Teniya 1 (T1) | 0 to 2 | Sept. 8, 2014 |  |  | XXXX | 2 |
| Teniya 4 (T4) | 0 to 2 | Sept. 8, 2014 |  |  | XXXX | 2 |
| Teniya 7 (T7) | 0 to 2 | Sept. 8, 2014 | XXXX | C1 | XXXX | 2 |
| Teniya 9 (T9) | 0 to 2 | Sept. 8, 2014 | XXXX | C1-related | XXXX | 1 |
| Teniya 16 (T16) | 0 to 2 | Sept. 8, 2014 | XXXX | C1 | XXXX | 2 |
| Teniya 19 (T19) | 0 to 2 | Sept. 8, 2014 |  |  | XXXX | 2 |
| Teniya 25 (T25) | 0 to 2 | Sept. 8, 2014 | XXXX | C1-related | XXXX | 1 |
| Uken 1 (U1) | 1 to 2 | Sept. 30, 2015 |  |  | XXXX | 1 |
| Uken 2 (U2) | 1 to 2 | Sept. 30, 2015 |  |  | XXXX | 1 |
| Uken 5 (U5) | 1 to 2 | Sept. 30, 2015 |  |  | XXXX | 1 |
| Mizugama 1 (MN1) | 0 to 2 | Oct. 17, 2015 |  |  | XXXX | 4 |
| Mizugama 3 (MN3) | 0 to 2 | Oct. 17, 2015 |  |  | XXXX | 2 |
| Mizugama S3 (MS3) | 0 to 2 | Oct. 17, 2015 |  |  | XXXX | 2 |
| Mizugama S4 (MS4) | 0 to 2 | Oct. 17, 2015 |  |  | XXXX | 2 |
| Mizugama S5 (MS5) | 0 to 2 | Oct. 17, 2015 |  |  | XXXX | 2 |
| Mizugama 6 (MN6) | 0 to 2 | Oct. 17, 2015 |  |  | XXXX | 1 |
| Mizugama S6 (MS6) | 0 to 2 | Oct. 17, 2015 |  |  | XXXX | 2 |
| Mizugama 7 (MN7) | 0 to 2 | Oct. 17, 2015 |  |  | XXXX | 2 |
| Kyan 2 (K2) | 1 to 2 | Oct. 2, 2015 |  |  | XXXX | 1 |
| Kyan 5 (K5) | 1 to 2 | Oct. 2, 2015 |  |  | XXXX | 1 |
| Kyan 7 (K7) | 1 to 2 | Oct. 2, 2015 | XXXX | C1-related | XXXX | 1 |
| Kyan 8 (K8) | 1 to 2 | Oct. 2, 2015 |  |  | XXXX | 1 |
| Odo H | 0 to 2 | Jan. 12, 2014 |  |  | XXXX | 1 |
| Odo L | 0 to 2 | Jan. 12, 2014 |  |  | XXXX | 1 |
| Odo M | 0 to 1 | Mar. 9, 2015 |  |  | XXXX | 1 |
| Odo N | 0 to 1 | Mar. 9, 2015 |  |  | XXXX | 4 |
| Odo O | 0 to 1 | Mar. 9, 2015 |  |  | XXXX | 1 |
| Odo P | 0 to 1 | Mar. 9, 2015 |  |  | XXXX | 1 |
| Odo T | 0 to 1 | Mar. 9, 2015 |  |  | XXXX | 1 |

*sequences too short to be included in alignment utilized in phylogenetic analyses.

***sensu* LaJeunesse (2001).

***see Materials and Methods for explanation of psbA^ncr^ lineages.
